# Does the medical insurance system play a real role in reducing catastrophic economic burden in elderly patients with cardiovascular disease in China? Implication for accurately targeting vulnerable characteristics

**DOI:** 10.1186/s12992-021-00683-7

**Published:** 2021-03-29

**Authors:** Meiyan Ma, Wanxin Tian, Jian Kang, Yuze Li, Qi Xia, Nianshi Wang, Wenqing Miao, Xiyu Zhang, Yiyun Zhang, Baoguo Shi, Han Gao, Tao Sun, Xuelian Fu, Yanhua Hao, Heng Li, Linghan Shan, Qunhong Wu, Ye Li

**Affiliations:** 1 Research Center of Public Policy and Management, School of Health Management, Harbin Medical University, Harbin, 150086 Heilongjiang China; 2 Heilongjiang Provincial Hospital, Harbin, Heilongjiang China; 3 Department of Medicine, Jiamusi University, Jiamusi, 154007 Heilongjiang China; 4 School of Ethnology and Sociology, Yunnan University, Kunming, Yunnan China; 5 Department of Economics, School of Economics, Minzu University of China, Beijing, China; 6 The Affiliated Tumor Hospital of Harbin Medical University, Harbin, Heilongjiang China; 7 Department of Health Service Management, School of Medicine, Hang Zhou Normal University, Hangzhou, Zhejiang China; 8 The Second Affiliated Hospital of Harbin Medical University, Harbin, Heilongjiang China; 9 Hospital Development institute of Shanghai Jiao Tong University, Shanghai, China

**Keywords:** Cardiovascular disease, Catastrophic health expenditure, Impoverishment by medical expense, Medical insurance, Health policy, China

## Abstract

**Background:**

The vulnerability of cardiovascular disease (CVD) patients’ health abilities, combined with the severity of the disease and the overlapping risk factors, leads such people to bear the economic burden of the disease due to the medical services. We estimated the economic burden of CVD and identified the weak link in the design of the medical insurance.

**Methods:**

Data from 5610 middle-aged and elderly with CVD were drawn from the 2015 wave of “*China Health and Retirement Longitudinal Study”* (CHARLS). The recommended method of the “*World Health Organization”* (WHO) was adopted to calculate “*catastrophic health expenditure”* (CHE), “*impoverishment by medical expenses”* (IME), and applied the treatment-effect model to analyze the determinants of CHE.

**Results:**

The incidence of CHE was 19.9% for the elderly families with CVD members, which was 3.6% higher than for uninsured families (16.3%). Families with CVD combined with > 3 other chronic diseases (38.88%) were the riskiest factor for the high CHE in the new rural cooperative medical system (NCMS). Moreover, families with members > 75 years old (33.33%), having two chronic disease (30.74%), and families having disabled members (33.33%), hospitalization members (32.41%) were identified as the high risky determinants for the high CHE in NCMS.

**Conclusions:**

Elderly with physical vulnerabilities were more prone to CHE. The medical insurance only reduced barriers to accessing health resources for elderly with CVD; however it lacked the policy inclination for high-utilization populations, and had poorly accurate identification of the vulnerable characteristics of CVD, which in turn affects the economic protection ability of the medical insurance. The dispersion between the multiple medical security schemes leads to the existence of blind spots in the economic risk protection of individuals and families.

## Highlights

- Older groups with physical vulnerability are more prone to catastrophic health expenditures.
- The medical insurance only guarantees the basic health utilization threshold for elderly with CVD, lacks policy inclination for high-utilization populations, and has poorly accurate identification of vulnerable characteristics, which in turn affects the economic protection ability of the medical insurance for patients with CVD.
- The unclosed connection between the multiple medical security systems leads to the existence of blind spots in the economic risk protection of individuals and families.

## Introduction

Aging, rapid urbanization, and shifting disease patterns are causing cardiovascular disease (CVD) to become established as one of the primary diseases in the worl d[1, 2]. Worldwide, roughly 17.9 million people die of CVD each year, and the absolute number of deaths from chronic CVD increased by 42.4% between 1990 and 2015 (2017 )[3, 4]. The incidence and mortality of CVD are also serious in China. As of 2015, 290 million people were suffering from CVD in China, including more than 9.5 million cases of heart disease [5]. Between 1990 and 2013, the total number of CVD deaths increased by 46% in China, mainly due to population aging [6]. The increasing trend of CVD has brought a heavy economic burden to the world at a global the population and householdlevel [7]. It is estimated that as of 2015, the estimated annual global cost of CVD is expected to increase by 16%, from $906 billion to more than $1 trillion by 2030 [8]. In India, 25% of families with a member with CVD experience catastrophic expenditure, and 10% are driven into poverty [9].

The particularity of the internal disease structure and the characteristics of the elderly have made China a country with a high incidence of CVD. In China, the disability-adjusted life years (DALY) caused by CVD has reached 582.255 million, accounting for roughly 20% of the world’s CVD, which is nearly 9% higher than the international average [10]. Annual inpatient care cost has risen at a steady rate of 30% since 2004 [11]. Compared to Western Europe, China suffers a 50% higher mortality rate from CVD and faces a larger economic burden [12]. Annual direct medical expenses for CVDs in China are more than 130 billion yuan, accounting for more than 22% of the total medical expenses in China during the same period, which is 10% higher than that of Australia [13]. Research indicates that during the 30 years from 2010 to 2040, the annual economic benefits of reducing cardiovascular mortality by 1% in China is equivalent to 68% of the real GDP in 2010, exceeding US$ 10.7 trillion [14].

China’s basic medical insurance system consists of three types of medical insurance schemes: urban employees’ insurance medical (MIUE), urban resident insurance medical (MIUR), and the new rural cooperative medical system (NCMS) [15]. The basic medical insurance for economic protection has two basic goals: first, to ensure that all people have access to high-quality care; and second, that everyone has the ability to pay for these services to maintain and improve their health [16]. The coverage of China’s medical insurance not only includes common diseases but also provides a certain extent of protection for sudden infectious diseases such as malaria and COVID-19 [17–20], which can reduce the family medical economic burden in multiple directions.

China has achieved 95% coverage of medical and health services. However, the inefficiency of the healthcare system and the uneven distribution of medical resources make the cost-effectiveness of continuous medical reforms lower, and the coverage and depth of the medical insurance system need to be improved. Studies have demonstrated that the actual effect of this coverage has been offset by the rapid escalation of medical expenses, especially for the elderly group that combines age and disease [21]. One study measured the disease burden of CVD patients aged 60 years and older in 2011–2013 and found that the direct and indirect medical costs of residents rose from 4938 yuan to 5717 yuan, and the out-of-pocket (OOP) increased by roughly 12 percentage points (from 58.1 to 70%) [22]. The resident’s medical insurance reimbursement ratio is substantially lower than that of the European Union’s (EU) major disease reimbursement ratio of not less than 60% [23]. The World Health Organization (WHO) reports that a reasonable OOP is 15–20% [24]. Once the literature exceeds the prescribed warning value, it will often cause vulnerable groups, such as rural residents and the elderly with low economic incomes, to fall into excessive cash health expenditures, resulting in “impoverishment by medical expenses” and “returning from poverty.”

These studies indicate that the vulnerability of CVD patients’ health abilities, combined with the severity and recurrence of the disease itself and the overlapping risk factors, leads them to bear the heavy economic burden of the disease due to the use of medical services. However, to our knowledge, the existing literature largely focuses on the whole population, and no studies target this particularly vulnerable group. Moreover, in previous studies, logistic regression was typically used to study the determinants of catastrophic health expenditure (CHE), ignoring the simultaneous causal relationship between potential unobservable variables and endogenous variables. To obtain more explanatory results, we applied the treatment-effect model and instrumental variable (IV) method to explore the influencing factors. We adopted the method recommended by the WHO to verify the health-reducing effect of the health care system, that is, whether the medical insurance system reduces the risk of CHE in cardiovascular patients over the age of 45 years. It aimed to capture the vulnerable characteristics that are prone to CHE and diagnoses the weak policy link that fails to eliminate CHE. It is important to understand whether the medical insurance system increases the accessibility and affordability of health care, which provides good evidence for further ways to alleviate health poverty.

## Methods

### Data source and sampling method

The data used to calculate the rates of CHE and impoverishment by medical expenses (IME) were obtained from the 2015 China Health and Pension Tracking Survey (CHARLS) database, which is designed to collect microscopic information from > 45-year-olds. CHARLS covers 450 communities in 150 counties from 28 of 32 provinces in mainland China. Face-to-face household interviews were conducted by qualified investigators. Households were randomly selected from maps and listings within each rural or urban community by four-stage stratified cluster sampling to select eligible individuals. Data were collected through questionnaires, and quality control was implemented by supervisors and included GPS comparison, data verification, recording verification, and telephone verification. A total of 5610 samples (2507 households) were finally obtained after excluding incomplete data and missing values.

### Statistical analysis

#### Calculation method for CHE and IME

The WHO’s recommended method was adopted to calculate CHE and IME [25]. CHE was defined as an OOP payment for health care equal to or exceeding 40% of a household’s capacity to pay. IME was defined as consumption expenditure equal to or higher than household subsistence expenditure but lower than the subsistence expenditure (SE) net of OOP health payments. The key expenditure indicators involved in the calculation process are as follows:

$$ \mathrm{Equivalent}\ \mathrm{family}\ \mathrm{size}: eqsiz{e}_h= hhsiz{e}_h^{0.56.} $$

Equivalent food expenditure: total household food expenditure divided by equivalent family size

$$ {eqfood}_h=\frac{foo{d}_h}{eqsiz{e}_h} $$

Poverty Line (pl): the weighted average food expenditure of a household, whose food expenditure as a share of household consumption expenditure fell between the 45th and 55th percentiles of the entire sample.

$$ pl=\frac{\sum {w}_h\ast eqfoo{d}_h}{\sum {w}_h} $$

Household subsistence expenditure (SE) was calculated using food expenditure as a share of total household consumption expenditure.

$$ s{e}_h= pl\ast eqsiz{e}_h $$

A household’s capacity to pay (CTP) was defined as non-subsistence spending of a household as a share of total household consumption expenditure.

$$ ct{p}_h={\exp}_h-s{e}_h\ \mathrm{if}\ {\mathrm{se}}_{\mathrm{h}}\le {\mathrm{food}}_{\mathrm{h}} $$

$$ ct{p}_h={\exp}_h-{food}_h\ \mathrm{if}\ {\mathrm{se}}_{\mathrm{h}}>{\mathrm{food}}_{\mathrm{h}} $$

Out-of-pocket (OOP) health expenditure: The payments made by households for their health services without third-party compensation.

$$ oopct{p}_h=\frac{oo{p}_h}{ct{p}_h} $$

Household consumption expenditure (exp) comprises both monetary and in-kind payments on all goods and services and the money value of the consumption of home-made products.

The consumption quintile is ranked by equivalized per capita household consumption expenditure weighted with the standard household size rather than the actual household scale.

$$ \mathrm{eq}\ \mathrm{ex}{\mathrm{p}}_h={\exp}_h/{\mathrm{eqsize}}_h $$

#### Treatment effect model

Relationship between participating in the medical insurance system and CHE is characterized by a joint causal relationship. There are implicit selection biases in participating in the medical insurance system, such as the average community participation rate, self-assessment of health status, and other unobserved characteristics associated with the initial selection. To address the bias caused by hidden selectivity bias and joint causality, we applied an IV method called the therapeutic effect model [26, 27].

#### Instrument indicators

We assume that the indicators of medical insurance are endogenous. To address this endogeneity, we required IVs, which are related to endogenous predictors (medical insurance) but are not related to the error term of outcome variables (CHE) [28]. The premise of using the IV method is that there are endogenous variables in the regression equation. Therefore, it is necessary to identify endogenous variables (whether there was participation in medical insurance) in the regression equation. In our study, we verify this problem using the DWH test. In other words, the DWH test checks whether the endogenous predictor is truly endogenous. (The heterogeneity test result shows that *P* = 0.000 < 0.05, so we use the DWH text)

By reviewing existing literature, two possible instruments were initially identified: self-assessment of health status and average community participation rate. Self-reported poorly healthy groups are more likely to choose to take medical insurance to maintain their health [29]. The average community participation rate of residents’ basic medical insurance will have an impact on the willingness of families to participate in insurance. People living in the same community have certain common characteristics, such as family economic level and health management awareness, which will make them participate in medical insurance. Moreover, the basic medical insurance plan in China is a community or company-based unit. For example, the MIUE is driven by the company, and the NCMS is that the village cadres mobilize the villagers to participate in the medical insurance scheme, so that the community participation rate is highly correlated with the willingness to participate in the family [30].

Good instruments should satisfy two main criteria: relevance and validity. That is, good instruments would be correlated with the endogenous variable (relevance criterion) but not with the error terms in the model of the outcome variable (validity criterion).

The overidentification test and GMM regression are used to check the validity and relevance [31, 32]. To further investigate the weak IV problem, we also performed redundancy tests. The results indicate that the community participation rate became our ultimate effective instrumental variable. The results regarding the validity and relevance are presented in Table 1.

**Table 1:** Validity test and correlation test

Instrumental variable	DWH test	Over-identification test	F test	Redundancy test
Community participation rate	chi2(1) =4.5588 (*p* = 0.0327 < 0.05) *	chi2(1) =1.6578 **(*p*** **= 0.1979 > 0.05)** *	**F = 24.3034 > 10**	Chi-sq.(1) = 0.0000*
Self-assessed health				Chi-sq.(1) =0.3873

DWH test, *p<0.05; Over-identification test, *p>0.05; Redundancy test, *p<0.05

The treatment effect model includes a two-stage regression. In the first phase, we regress the outcome variables of the covariates (the heterogeneity test results reveal that *P* = 0.000 < 0.05, so we use ‘Generalized method of moments’ (GMM) as our regression equation). Based on the results of the first phase, we add instrument variables and the outcome variable performs a quadratic regression.

#### Outcome variable

We created a binary indicator for CHE as the outcome variable (1 = occurrence, 0 = no occurrence).

#### Covariates

In our study, we used three sets of information as covariates: (1) social-demographic characteristics, specifically age, gender, marital status, education level of household head, family size, and having a member over 65 years old; (2) health status of family members, including whether the household has at least one member with a chronic disease, who has been admitted to the hospital, or has a disability; (3) demand and utilization of health services for household heads, mainly including hospitalization rate, outpatient rate, and non-admission rate (defined as the percentage of patients who need to be hospitalized but are not hospitalized).

## Results

### Sample characteristics

Table 2 summarizes the characteristics of CVD in 2015. The total sample comprised 2507 households and 5610 individuals, of which 50.2% of household heads with CVD were male, and around 65.7% of the elderly were aged < 64. Over 59.4% had an elementary to junior high education level, only 23.3% of CVD patients had a health satisfaction status of “good,” with > 60.4% having one or more other chronic diseases. Furthermore, 97.1% of the CVD patients had medical insurance.

**Table 2:** Patients’ demographic characteristics

Variables	Variable value	Percentage (%)
CHE	No	80.1
	Yes	19.9
Participate in medical insurance scheme	No	2.9
	Yes	97.1
Gender of household head	Female	49.8
	Male	50.2
Marital status of household head	Others	15.0
	Married	85.0
Education level of household head	Illiteracy	21.5
	Elementary to junior high	59.4
	High school and above	15.4
Family size	1	62.6
	2–3	31.9
	More than 3 people	5.5
Age of household head	45–54	32.5
	55–64	33.2
	65–74	23.5
	More than75years old	8.7
Having members over 65 years old	No	44.2
	Yes	54.0
Combined with other chronic diseases	No	39.6
	1	35.5
	2	21.3
	3	3.6
Having hospitalized members	No	87.1
	Yes	12.2
Having disabled members	No	3.1
	Yes	92.3
Health satisfaction	Very good	4.2
	Good	19.1
	Fair	48.8
	Poor	20.5
	Very poor	6.8
Household consumption per capital quintile	Lowest	20.7
	2	21.3
	3	19.9
	4	20.2
	Highest	17.9
Region	Eastern	33.7
	Middle	46.3
	Western	20.0
location	Rural	75.1
	Urban	24.9

### Health care needs and service utilization (Table 3)

Regarding health service utilization for the high-risk group of elderly patients with CVD, the rate of monthly prevalence was 11.4% overall. A higher proportion of patients with CVD used inpatient services (14.8%), and more than 19.8% of respondents reported the utilization of outpatient services over the past month. The above indicators demonstrate different trends for different populations with CVDs as follows (Table 3):

**Table 3:** Health care demand, service utilization, and reimbursement of household head

	Prevalence (%)	Hospitalization rate (%)	Outpatient rate (%)	Non-admission rate (%)	Hospitalization reimbursement ratio (%)
Family size					
1	14.1	15.6	19.8	6.3	41.1
2–3	13.6	13.5	19.3	5.7	52.8
More than 3 people	13.8	14.7	24.0	8.3	49.1
Hospitalization members					
Yes	24.7	/	34.2	13.1	44.6
No	12.3	/	17.3	5.0	/
Having disabled members					
Yes	21.7	17.2	21.8	9.9	40.3
No	13.4	14.6	19.6	5.8	46.0
Combined with other chronic diseases					
No	12.0	13.3	17.7	5.3	45.3
1	14.0	15.3	20.8	6.0	46.8
2	16.6	14.6	20.0	7.4	49.1
≧3	13.8	23.5	24.5	9.8	48.5
Medical insurance schemes					
MIUE	8.6%	20.4	20.0	3.3	69.8
MIUR	15.5	18.4	19.1	8.5	52.0
NCMS	14.3	13.7	20.5	6.6	39.7
Integrated insurance	14.7	16.3	23.3	4.7	54.2
Others and not having insurance	13.0	13.5	14.3	7.2	59.2
Economic quintile					
Lowest	13.1	14.9	19.9	6.3	41.8
2	12.4	14.8	19.5	6.2	42.5
3	14.2	13.4	19.6	5.2	38.3
4	15.8	14.8	19.6	6.8	42.7
Highest	14.0	16.4	20.9	6.5	49.2
Overall	11.4	14.8	19.8	6.2	44.5

Cardiovascular patients with a high level of health service demand but low utilization of health services and low reimbursement rates. For example, families with CVD and > 3 other chronic diseases had a high demand for health services. Its prevalence and hospitalization rates were 13.8 and 23.5%, respectively. However, the health service utilization (non-admission rate 9.8%) and the reimbursement level (48.5%) were lower than those of CVD patients without other chronic diseases.

In terms of family economic level and type of medical insurance, cardiovascular patients with a high level of health service demand and high health utilization but low reimbursement. Household heads of CVDs with NCMS had a higher prevalence (14.3%) and outpatient rate (20.5%), but the hospitalization reimbursement ratio (39.7%) was lower by nearly 30% than MIUE (69.8%). Patients who participated in MIUR of CVDs demonstrated a similar pattern. Notably, among the wealthiest households with CVDs, the hospitalization and visit rates were 16.4 and 20.9%, respectively, but the hospitalization reimbursement ratio was only 49.2%, less than 50%.

### CHE and IME in different households (Table 4)

We further measured the incidence of CHE and IME in elderly patients with CVD, and 19.9% of all interviewed CVD patients encountered CHE, and the IME occurred in 7.6% of the overall population (Table 4).

**Table 4:** CHE, and IME in different households

	Incidence of CHE (%)	Incidence of IME (%)	OOP/CTP (%)
Family size			
1	22.6	8.6	21.1
2–3	15.6	6.2	13.7
More than 3 people	14.4	3.6	12.8
Hospitalization members			
Yes	29.3	8.7	28.4
No	18.6	7.5	15.6
Having disability members			
Yes	31.1	10.3	35.6
No	19.5	7.4	16.7
Health satisfaction			
Very good	17.1	4.1	17.0
Good	17.3	5.8	17.5
Fair	17.5	7.7	14.1
Poor	24.4	8.5	21.7
Very poor	30.5	10.0	25.3
Combine other chronic diseases			
No	14.7	6.1	14.6
1	20.2	6.8	18.8
2	27.0	10.5	19.2
≧3	31.8	14.2	22.8
Medical insurance			
MIUE	13.3	3.1	13.0
MIUR	15.1	4.5	13.8
NCMS	21.6	9.0	19.7
Integrated insurance	20.5	5.1	6.1
Others and not having insurance	16.3	3.8	15.3
Household consumption per capital quintile			
Lowest	19.3	8.3	17.9
2	22.5	10.1	22.1
3	20.4	7.8	18.4
4	19.5	5.9	16.2
Highest	17.5	5.5	13.6
Overall			
/	19.9	7.6	17.2

We also found that as the family size increased for CVD patients, the risk of households being trapped in CHE and IME was reduced. For example, the incidence of CHE among households with one person (8.1%) was twice that of those without other diseases (14.7%). Furthermore, families with inpatients and disability members of CVD increased the risk of CHE and had a greater financial burden, and the incidence of CHE was 29.3 and 31.1%, which was more than 10% higher than that of those without inpatients and disability members of CVD (18.6 and 19.5%, respectively).

Income level was not the main reason for the high incidence of CHE in patients with CVD. Among them, sub-poor families with CVD had the highest incidence rate of CHE (22.5%) and health expenditure burden (22.1%), which was higher than the richest households with CVD. Regarding the type of insurance, among the three basic medical insurances, cardiovascular families with NCMS had the greatest economic burden of disease, and the risk of CHE was also the highest (21.6%), nearly 8.3% higher than that of NIUE (13.3%).

### Treatment-effect model for patients with CVD (Table 5)

Community participation rate was added as an effective instrumental variable to the treatment-effect model. The results were as follows: family size, health satisfaction, combination with other chronic diseases, having hospitalization members, having disabled members, and participating in insurance were all found to be significantly associated with the odds of encountering CHE with CVD (*P* < 0.05).

**Table 5:** Results of treatment-effect model for patients with CVD

	Coef.	Std. Err.	z	***P*** > |z|	95% C.I.	
**CHE**						
Marital status of household head	0.0129	0.0233	0.53	0.599	−0.0335	0.0581
Region	−0.0209	0.0116	−1.79	0.073	−0.0438	0.0019
Gender of household head	0.0081	0.0174	−0.47	0.640	−0.0424	0..0261
Education level of household head	0.0016	0.0151	0.11	0.915	−0.0281	0.0313
Household consumption per capital quintile	−0.0069	0.0061	−1.12	0.262	−0.0190	0.0052
**Family size**	**−0.0499**	**0.0138**	**−3.61**	**0.000**	**−0.0770**	**−0.0227**
Urban and rural	−0.0379	0.0203	−1.86	0.063	−0.0779	0.0019
**Health satisfaction**	**0.0246**	**0.0093**	**2.64**	**0.008**	**0.0063**	**0.0429**
**Having disabled members**	**0.0961**	**0.0473**	**2.03**	**0.042**	**0.0033**	**0. 1889**
Having members over 65 years old	0.0129	0.0171	0.76	0.450	−0.0206	0.0465
**Combined with other chronic diseases**	**0.0468**	**0.0088**	**5.32**	**0.000**	**0.0296**	**0.0641**
**Having hospitalized members**	**0.1001**	**0.0252**	**3.97**	**0.000**	**0.0506**	**0.1495**
**Having insurance**	**0.2895**	**0.1454**	**1.99**	**0.047**	**0.0044**	**0.5745**
**Having insurance**						
**Community participation rate**	**0.0571**	**0.0056**	**10.08**	**0.000**	**0.0460**	**0.0682**

*Coef* Coefficient, *Std. Err* standard error of the mean, *95% C.I* Confidence Interval

#### Social demographic perspective

The incidence of CHE and family size with CVD have developed in reverse: as the cardiovascular family grows in size, the risk of CHE is reduced by 4.99 percentage points.

#### Health needs and utilization perspective

Combined with other chronic diseases, health satisfaction**,** having hospitalization members, and having disabled members had a positive correlation with the risk of CHE in cardiovascular patients, and with an increase in combining other chronic diseases or hospitalization members increased, the risk of a family’s CHE increased by 4.68 and 10.01%, respectively.

#### Medical insurance perspective

Cardiovascular patients participating in medical insurance increased the risk of CHE and increased the risk of being trapped in CHE by 28.9%.

### Medical insurance level

**Fig. 1:**
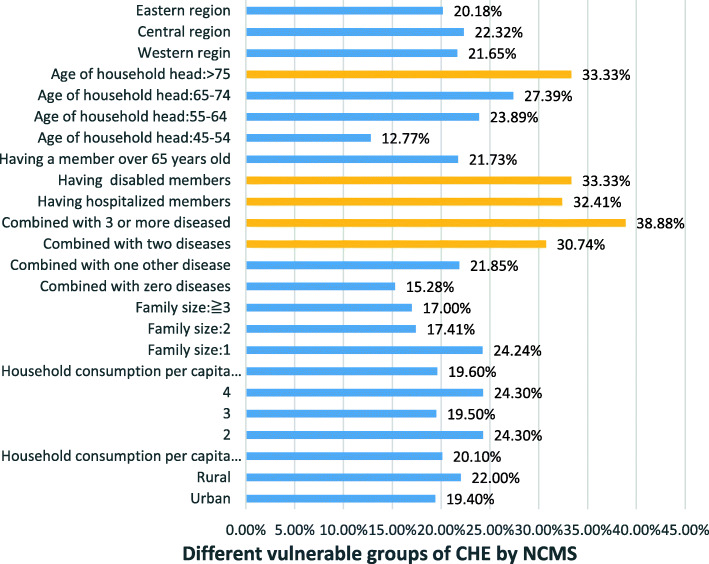
Different vulnerable groups of CHE by NCMS

As shown in the above figure, among patients with CVD, the rural population accounted for 75.1% of the sample size, and the CHE of NCMS was the riskiest, at 21.6%. Moreover, the treatment-effect model demonstrates that the probability of CHE participating in health insurance families with CVD has increased by 29.8%. Therefore, to identify the NCMS of key bottlenecks, we conducted a specific influencing factor analysis and found that families with CVD combined with > 3 other chronic diseases (38.88%) were the primary cause of high CHE in NCMS. Next, in turn, being more than 75 years old (33.33%), having disabled members (33.33%), having hospitalization members (32.41%), and combining two chronic diseases (30.74%) were the top five factors influencing the high CHE of families with CVD in NCMS. The results are presented in Fig. 1.

## Discussion

The proportion of households associated with CHE and IME in elderly patients with CVD were 19.9 and 7.6%, respectively. These rates were higher than those of developed countries but were lower than those of low-income countries [33, 34]. The incidence of CHE participating in medical insurance was 3.6% higher than that of uninsured households (16.3%). Families with CVD have a substantially higher risk of IME than families without CVD (7.0%) and the overall population (7.2%). CVD patients’ risk tolerance for health care payments is lower than the average in China. Simultaneously, our study also found that cardiovascular families with chronic patients, inpatients, and disabled members are at a higher risk of falling into poverty because of the cost of health care, and they are becoming a stubborn group with a high burden of CVD under the medical insurance system.

As a vulnerable group, patients with CVD are mainly characterized as a high-risk group that integrates physiological, social, and health factors. It is important to identify the risk factors for patients with CVD and identify vulnerable groups to better play the economic protection role of the medical insurance system. As mentioned earlier, despite these government efforts, many disadvantaged groups are not considered target populations for benefit enhancement. Through comprehensive analysis, we found that the disadvantaged elderly population with mental health has the following characteristics:

### Older groups with physical vulnerability are more prone to CHE

First, age growth, loss of healthy capital, and decline in physiology are inevitable for the elderly. However, elderly people are at a disadvantage in accessing resources and fail to enjoy social welfare policies fairly [35, 36]. For example, the incidence of CHE in elderly people aged over 75 years in the NCMS was 33.33%, second only to the merger of three chronic diseases (33.88%). The WHO reported that 23% of the world’s disease burden is on older people, and chronic non-communicable diseases have a major impact on this burden [37]. Furthermore, the morbidity and concurrency of elderly patients with CVD may eventually lead to premature death and disability, while long-term health care costs, drug costs, and rehabilitation cost greatly increase the risk of CHE [38]. Our results indicate that the hospitalization rate (23.5%), rate of visits (24.5%), and incidence of CHE (31.8%) of patients with more than three chronic diseases are far higher than those without chronic diseases (13.3, 17.7, 14.7%, respectively), and the treatment-effect model showed that the combination with other chronic diseases increased the risk of CHE by 4.68%. Past evidence has demonstrated that the likelihood of using health care (e.g., hospitalization) increases in the presence of chronic or multiple conditions [39]. With the increase in the number of chronic diseases, the addition of other chronic diseases will prolong the hospitalization time on the basis of the original single disease, which will cause CVD patients to superimpose the cost of other diseases when they bear the economic pressure from their CVD burden. Studies have found that 1/3 of adults have multiple chronic diseases, equivalent to 3/4 of the elderly in developed countries [40]. Therefore, suffering from a variety of chronic diseases has become a major health problem for the elderly in the future, greatly increasing the risk of CHE.

### The medical insurance system only guarantees the basic health utilization threshold for elderly patients with CVD and lacks policy inclination for high-utilization populations

The existing medical insurance system reduces the threshold of health service utilization for vulnerable groups. Such as those with CVDs, but it only achieves the first goal of medical insurance—that is, to ensure that all people have access to high-quality care. Our results indicate that the incidence of CHE for high-demand people with inpatients and disabled patients is substantially higher than that of the normal population. In our study, families with disabled patients had a higher prevalence (21.7%) and outpatient rates (21.8%) than those without disabilities (13.4 and 19.6%, respectively). However, the hospitalization reimbursement ratio was only 40.6%, far below the overall level (44.5%), and OOP accounted for 35.6% of total household health expenses, which is substantially higher than that of OECD countries [41]. A study in South Korea revealed that families with disabilities face higher CHE than those without people with disabilities, and annual living expenses for OOP medical expenses are roughly 1.2 to 1.4 times greater [42]. This may be due to physical or mental disability in disabled patients leading to job loss or reduced earnings, while higher medical care needs due to disability increase the burden of high medical costs [43]. Even relatively small expenditures are catastrophic for poor families, and excessive OOP health care spending can lead to poverty [44].

### The medical insurance system has poorly accurate identification of vulnerable characteristics, which in turn affects the economic protection ability of the medical insurance system for patients with cardiovascular diseases

China’s medical insurance policy aims to solve the problem of “people falling into poverty due to illnesses” and to ensure that most people are not reduced to poverty because of health-related issues. However, our results indicate that the risk of CHE for CVD patients participating in medical insurance schemes has increased by 28.9%, which had the highest incidence of CHE for NCMS. The medical insurance system has the disadvantage of insufficient protection of policies in reducing the economic burden of residents’ medical care and maintaining residents’ health rights and health. China’s health sector reform has achieved unprecedented progress, but protecting vulnerable groups from healthcare-related impoverishment remains a challenge. The goal of full coverage has been achieved, and the financing level of NCMS has improved. By 2019, the annual premium increased from 30RMB (US$3.62) in 2003 to 740 RMB (US$105.7) per person, with 520 RMB (US$74.3) from central and local governments, and 220 RMB (US$31.4) from households [45]. Although the reimbursement rates of NCMS vary across counties and especially provinces, its reimbursement level of the scheme has significantly improved since the initial stage [46]. However, for the poor, the elderly, the patients with CVD, or the groups with multiple vulnerabilities, the improvement in risk pooling and government subsidies of NCMS may be offset by the increasing medical expenses [47, 48]. Several scholars also called for a more generous package to improve the security capacity of the NCMS pursuing the CHE and IME reduction in the short term [49]. However, simply increasing the reimbursement rate is not feasible in the long-term as it may lead to the insolvency of the medical insurance fund and have a more long-term negative impact on the insured members [50]. Specifically, it is important to adopt a comprehensive governance strategy that includes reconsidering benefit packages, redesigning social health care insurance programs, and controlling the price of medical services to further protect vulnerable groups such as the elderly population with CVD.

#### Inequality between types of health insurance systems: binary urban and rural structure divided by place of residence

The household registration system in China directly affects the ability to obtain various medical benefits [51]. In our study, the hospitalized reimbursement ratio of MIUE with CVD patients was 69.8%, while that of the NCMS was only 39.7%, accounting for only half of the MIUE. Urban residents are expected to have a greater awareness of their health and better access to health insurance (especially private health insurance) and hence are more likely to obtain health insurance. The health needs of people in rural areas are not able to be converted into effective medical needs in time due to lower income levels, high medical prices, and inadequate medical care, thus bearing the risk of greater CHE. A Chinese study demonstrates that NCMS cannot prevent CHE from happening in poor families but only reduces its incidence in wealthy families [52]. This differentiated design of health care benefits can sometimes lead to social inequalities, often with the same disease, at different costs, and patients with higher socioeconomic status usually enjoy better health insurance and higher service utilization. In rural areas, this should further increase the reimbursement ratio of outpatient and inpatient expenditures to the elderly with CVDs who receive treatment at every kind of hospital [53].

### With the inheritance of the traditional concept of supporting the elderly in China, the larger family size is the protective factor for CHE

First, our results reveal that family size is a protective factor, that is, the larger the family size, the lower the risk of suffering catastrophic health expenditure. This finding is in line with other studies [54, 55].

People with CVD can hardly be social workers, which leads to the loss of labor ability. Smaller families lead to less labor, which will decrease the family’s ability to resist economic risks and further result in CHE [56, 57]. Vulnerable individuals with a chronic disease not only have poor financial ability but also pose a health economic burden to their families. More family members make the individual share less.

Second, the concept of family and filial piety is deeply rooted in China’s traditional virtue. Supporting the elderly is the responsibility and obligation, which is written in Chinese law. This kind of social culture deeply influences the economic decisions of Chinese families. Families with more members will provide more social and financial support to resist the dilemma of catastrophic health expenditure.

#### Inequality of different income groups under the same medical insurance system

Our study indicated that the incidence of CHE in sub-poor households with CVD (22.5%) was higher than 5% of households with the highest income. Its OOP accounts for 22.1% of CTP, and the economic burden of disease was higher than that of high-income families by nearly 10%. A similar phenomenon occurred in India. According to the data, India’s medical expenditure accounts for only 0.9% of GDP, which is lower than the 2.8% of the GDP of less developed countries. However, with only government spending, among the five economic subgroups, the poorest households with less income received only 10% of medical care, while 20% of the wealthiest households receive up to 33% of social subsidies, three times as substantially as the poorest households [58]. The significant burden of disease can lead to difficult family life in low-income groups, which thus fall into the evil cycle of “poor illness due to illness and illness due to poverty.”

Poor families are at a greater risk of suffering CHE than well-off families and wealthiest families who receive more medical care subsidies [59–61]. It is unfair to poor families to implement the policy of non-differentiated compensation level and welfare package coverage. The capacity to pay for families with different economic levels should be taken as an important factor in formulating compensation strategies. The key link in the design of medical insurance schemes should be inclined to the poor, such as reducing the threshold of outpatient and inpatient deductible for the poor, expanding the coverage of major disease and chronic diseases, and even helping poor families to pay premiums through medical assistance. Therefore, regardless of the type of medical insurance scheme, priority should be given to the differences in compensation and benefit coverage for families with different economic levels during the process of top-level design of the medical insurance system.

### Inconsistencies between multiple health care systems lead to blind spots in the economic risk protection of individuals and families

China has established multiple medical security systems, including basic medical insurance, major illness insurance, commercial medical insurance, social medical assistance, and charity assistance. However, the complexity of CVDs and the difference in the income of the population means that various systems have not woven a standard, unified safety net to prevent impoverishment by medical expenses, which is mainly reflected in the following two points.

First, the particularity of CVD can lead to the loss of labor, the indirect cost of nursing expenses, and transportation expenses, and long-term drug maintenance will also increase the economic burden of patients. These neglected indirect costs and drug costs have become blind spots in the economic protection of basic healthcare systems. In our study, elderly cardiovascular patients still have to pay more than half (55.5%) of their medical expenses after reimbursement by basic medical insurance schemes. Moreover, there is no effective mechanism to connect basic insurance schemes, major disease schemes, and medical assistance insurance systems, resulting in this group of patients falling into poverty after paying for high medical expenses.

Second, our results also show that sub-poor households have the highest incidence of CHE at 22.5%, and the OOP accounts for 22.1% of CTP, substantially higher than the poorest households. The medical security system has implemented certain health expense reduction policies for these “extremely poor families” without economic sources or micro-income, including lowering the deductible line, increasing the proportion of reimbursement, and improving or eliminating top-up measures. The high-risk marginalized population with an economic income slightly higher than that of the poorest households, which is prone to CHE, has not reached the standard of assistance for supplementary medical assistance and has failed to be covered by a multilevel medical security system.

It is evident that exploring the integration of multilevel medical insurance systems, promoting the complementary functions and overlapping effects of multilevel medical insurance systems, and jointly solving the poverty problems caused by cardiovascular patients have become the target task of the current prevention and treatment process of chronic diseases.

## Conclusions

Our study findings reveal that the incidence of CHE and IME was relatively high among elderly households with CVD patients. The elderly who had poor health satisfaction, combined with other chronic diseases, inpatients, and disabled people, were the high-risk groups for CVD. Medical insurance programs have not reduced the risk of CHE or relieve the financial burden of CVD patients in China, especially the elderly who participate in the NCMS. Therefore, there is an urgent need for Chinese policymakers to improve the accuracy of medical health insurance schemes to target the vulnerable characteristics of elderly cardiovascular patients. While improving the accessibility of health services for cardiovascular patients, the next step should be to focus on achieving another function of the medical health insurance system, which is to maximize the economic protection for vulnerable enrollers and reduce their risk of being trapped in poverty, rather than increasing the risk. It is worth noting that our research has its limitations. First, the data were self-reported, which may make it prone to measurement errors. Second, the study used cross-sectional samples rather than panel data, making it difficult to test the long-term effects of health insurance and other control variables on CHE in cardiovascular patients.

## Data Availability

The datasets generated during and/or analyzed during the current study are available in the CHARLS repository, [http://charls.pku.edu.cn/pages/data/111/zh-cn.html]
